# Hepatic Expression of Metallothionein I/II, Glycoprotein 96, IL-6, and TGF-**β** in Rat Strains with Different Susceptibilities to Experimental Autoimmune Encephalomyelitis

**DOI:** 10.1155/2013/750406

**Published:** 2013-12-04

**Authors:** Tanja Grubić-Kezele, Gordana Blagojević Zagorac, Hrvoje Jakovac, Robert Domitrović, Čedomila Milin, Biserka Radošević-Stašić

**Affiliations:** ^1^Department of Physiology and Immunology, Medical Faculty, University of Rijeka, B. Branchetta 22, 51 000 Rijeka, Croatia; ^2^Department of Chemistry and Biochemistry, Medical Faculty, University of Rijeka, B. Branchetta 22, 51 000 Rijeka, Croatia

## Abstract

In a search of peripheral factors that could be responsible for the discrepancy in susceptibility to EAE in Albino Oxford (AO) and Dark Agouti (DA) rats, we estimated the expression of metallothioneins I/II (MT), heat shock protein-gp96, interleukin (IL)-6, and transforming growth factor (TGF)-**β** in the livers of these animals. Rats were immunized with bovine brain homogenate (BBH) emulsified in complete Freund adjuvant (CFA) or only with CFA. Western blot and immunohistochemical analyses were done on day 12 after the immunization, as well as in intact rats. The data have shown that during the first attack of EAE only the EAE prone-DA rats markedly upregulated the hepatic MTs, gp96, IL-6, and TGF-**β**. In contrast, AO rats had a significantly higher expression of MT I/II, IL-6, and TGF-**β** in intact liver (*P* < 0,001), suggesting that the greater constitutive expression of these proteins contributed to the resistance of EAE. Besides, since previously we found that AO rats reacted on immunization by an early upregulation of TGF-**β** on several hepatic structures (vascular endothelium, Kupffer cells, and hepatocytes), the data suggest that the specific hepatic microenvironment might contribute also to the faster recovery of these rats from EAE.

## 1. Introduction

Multiple sclerosis (MS) is a heterogeneous disease in which different mechanisms, such as an autoimmune attack, inflammation, neurodegeneration, and intoxication, induce demyelination, loss of oligodendrocyte and neurons, and axonal injuries. The triggering event is usually the invasion of peripherally activated myelin-specific Th1 and Th17 immune cells in CNS, where they interact with antigen presenting cells (APC) and microglial cells that drive the inflammatory cascade leading to tissue damage and an amplification of the initial immune reaction [1–3]. Underlying mechanisms include, therefore, the breakdown of tolerance to autoantigens, as well as the activation of cascades of cellular and molecular events that contribute to initiation of the injury or infection, such as the production of proinflammatory cytokines, chemokines, and cell adhesion molecules as well as the release of new collateral factors, such as damage-and pathogen-associated molecular patterns (DAMPs and PAMPs) that may potentiate the injury [4]. The outcome depends also on the activation of local and systemic cytoprotective and anti-inflammatory mechanisms, as well as on the interplay between the pathogenic and regulatory T (Treg) cells subpopulations that modulate the autoimmune attack. The pathogenesis is multifactorial and dependent also on the interrelationship between the neuroendocrine and the immune system [5, 6], as well as on the presence of genes that determine both the immune functions and the target organ susceptibility for autoimmune disease [7].

Regarding the genetic background of EAE, a large number of information was obtained also in DA and AO rats' strains, which have different susceptibilities to EAE [8–11].

Thus, the discrepancy was found in the numbers of immune cells that immigrate in the CNS, in production of IL-2, interferon (IFN)-gamma, and interleukin (IL)-17 as well in the secretion of IL-6, TGF-beta, and IL-10 within the CNS or in draining lymph node cells. Contributing to this field we have recently shown that these rat strains differ also in the activation pattern of metallothioneins I/II [12], which within the mammalian CNS perform essential cytoprotective functions, owing to their metal binding, antioxidative, antiapoptotic, and growth-regulatory activities [13–16]. In these data [12], we have shown that constitutive and induced MT I+II gene expression in EAE-resistant and EAE-prone rats is different both in the organs that were damaged by the autoimmune attack (hippocampus and cerebellum) as well as in the liver, pointing to the high involvement of the central and peripheral MTs-related mechanisms in the induction of EAE. Besides, since in AO rats, early after immunization with encephalitogen (on the seventh postimmunization day), we found a marked upregulation of TGF-beta immunoreactivity on several hepatic structures, we hypothesized that immunosuppressive environment in the liver probably contributed also to the induction of resistance toward EAE [12].

In an attempt to enlarge these data, in DA and AO rats, we made a quantitative analysis of hepatic expression of MT-I/II proteins and two hepatic cytokines (IL-6 and TGF-*β*), which participate in acute phase reaction and in the creation of the specific hepatic cytokine microenvironment that governs the local balance between tolerance and immunity [17]. Moreover, owing to emerging evidence indicating that critical regulators of cell stress response in neurological diseases [18] as well as in the immune response [19–21] are the heat shock proteins (HSPs), in DA and AO rats we estimated also the hepatic expression of endoplasmic reticulum (ER) resident HSP-gp96, which according to our own data might have the essential function in the pathogenesis of chronic relapsing EAE in DA rats [22].

The data have shown that only EAE-prone DA rats significantly upregulated the hepatic MTs, gp96, IL-6, and TGF-*β* during the appearance of first clinical symptoms, as well as that AO rats have significantly higher MT I+II, IL-6, and TGF-*β* expression in intact liver, suggesting that a high constitutive expression of these proteins might contribute to the resistance of EAE.

## 2. Materials and Methods

### 2.1. Experimental Animals

For the experiments male, Dark Agouti (DA), and Albino Oxford (AO) rats were used, aged 2-3 months. They were bred and maintained according to the guide for Institutional Animal Care and used with approval of the Local Ethical Committee.

### 2.2. EAE Induction

Immunization was performed by bovine brain white matter homogenate emulsion (BBH) in the complete Freund's adjuvant (CFA) (Sigma, St. Louis, MO, USA), as we previously described [22–24]. Each animal received 2 × 0.1 mL of the emulsion, which was injected subcutaneously, in each hind footpad. Control group was injected with the same dose of CFA. Animals were sacrificed on the 12th day after immunization with appearance of heavy clinical symptoms of EAE that involve hind legs paralysis with incontinence. The severity of disease was clinically assessed according to the following criteria: 0: no symptoms; 1: faccid paralysis of tail; 2: hind legs paresis; 3: hind legs paralysis with incontinence, and 4: death of animal.

### 2.3. Antibodies and Reagents

Mouse monoclonal antibody to MT I/II (clone E9) was obtained from Dako Cytomation, USA, and diluted 1 : 250 for WB and 1 : 50 for IHC-P. Monoclonal Rat IgG2a antibody to Grp94/gp96 (clone 9G10) was obtained from Stressgen, Canada, and used in dilution 1 : 1500 for WB and 1 : 100 for IHC-P. Mouse anti-rat antibody to IL-6 was obtained from R&D systems, Abingdon, UK, and used in dilution 1 : 200. Rabbit polyclonal IgG antibody to TGF-*β* 1 (1 : 600 for WB and 1 : 100 for IHC-P) was obtained from Abcam, Cambridge, UK. Horse reddish peroxidase (HRP)-goat anti-mouse IgG and HRP-goat anti-rat antibodies were obtained from Jackson ImmunoResearch Laboratories, Inc., USA. HRP-goat anti-rabbit conjugated antibody, ECL Prime Western Blotting Detection Reagent, and Hybond ECL nitrocellulose membrane were obtained from GE Healthcare, Uppsala, Sweden. Al HRP secondary antibodies were used in dilution 1 : 10 000. Goat anti-mouse *β*-actin antibody was obtained from Santa Cruz Biotechnology, Inc., USA, and used in dilution 1 : 2000.

### 2.4. Western Blot Analyses

Equal amounts of frozen liver tissues were homogenized and lysed in radioimmunoprecipitation assay (RIPA) buffer containing 25 mM Tris-HCl pH 7.6, 300 mM NaCl, 1% NP40, 1% sodium deoxycholate, 0.1% SDS, 2 mM PMSF, and complete protease inhibitor cocktail tablets obtained from Roche, Mannheim, Germany. Proteins were separated by 13% sodium dodecyl sulfate polyacrylamide gel electrophoresis (SDS-PAGE), under nonreducing conditions and then transferred onto a nitrocellulose membrane (GE Healthcare, Uppsala, Sweden). Membranes were then blocked with 1% blocking reagent (Roche Diagnostics GmbH) in tris-buffered saline with 0.5% Tween-20 (TBST) and then incubated with different primary Abs, followed by incubation with species specific HRP-conjugated secondary antibodies. The *β*-actin was used as a control of protein loading. Membranes were washed three times with TBS buffer, incubated with Amersham ECL Prime (GE Healthcare, Uppsala, Sweden), and scanned with Kodak Image Station 440CF (Kodak, New Haven, CT, USA). The intensity of the bands was quantified using ImageJ software (http://rsb.info.nih.gov/ij/). To determine the relative expression of analyzed proteins in liver tissue of immunized animals and animals treated only with CFA, the band density of each liver sample was compared with the band intensity obtained from liver tissue of intact animals after normalization to an internal control (*β*-actin). The analyses were made in tissue samples made as a pool from 3 rats in three separate experiments.

### 2.5. Tissue Preparation for Paraffin Slices

Liver were rapidly removed and fixed in 10% buffered formalin solution during 24 h. Tissue was then embedded in paraffin wax and sections were cut at 4 *μ*m using HM 340E microtome (Microtom, Germany). Heat induced epitope retrieval was done prior to staining procedure by heating tissue slides in boiled citrate buffer pH 6.0 four times, each 5 minutes, using a microwave steamer.

### 2.6. Immunohistochemistry

Immunohistochemical studies were performed on paraffin embedded liver tissue slides using DAKO EnVision + System, Peroxidase (DAB) kit according to the manufacturer's instructions (DAKO Corporation, USA). Briefly, slides were incubated with peroxidase block to eliminate endogenous peroxidase activity. After washing, antibodies were added to tissue samples and incubated overnight at 4°C in a humid environment, followed by 45 min incubation with peroxidase labeled polymer conjugated to goat anti-mouse or anti-rabbit immunoglobulins containing carrier protein linked to Fc fragments to prevent nonspecific binding. The immunoreaction product was visualized by adding substrate-chromogen (DAB) solution. Tissues were counterstained with hematoxylin, dehydrated trough graded ethanols, mounted using Entelan (Sigma-Aldrich, Germany), and examined with Olympus BX51 microscope (Olympus, Tokyo, Japan). The specificity of the reaction was confirmed by substitution of primary antibodies with isotype matched irrelevant immunoglobulins, used under the same conditions and dilutions as primary antibodies.

### 2.7. Statistical Analysis

Data were expressed as mean ± SE. Differences between groups were assessed by Friedman one-way analysis of variance (ANOVA), by Mann-Whitney *U* test and by two tailed Student's *t*-test. The level of significance was set at *P* < 0.05.

## 3. Results

As we previously described, the AO rats after immunization with BBH+CFA did not exhibit any clinical symptom of disease, in contrast to genetically susceptible DA rats, which develop a typical chronic-relapsing form of EAE (CR-EAE). In this study, animals from both groups were sacrified on day 12 after the immunization, that is, at time of the appearance of first attack of disease in DA rats. Protein content of MT I/II, gp96 and cytokines was analyzed in hepatic tissue samples obtained from immunized DA and AO rats (*N* = 9), from rats treated with CFA (*N* = 9), and from untreated rats (*N* = 9).

### 3.1. Hepatic Expression of MT I+II Proteins in EAE-Resistant and EAE-Prone Rats

**Figure 1:**
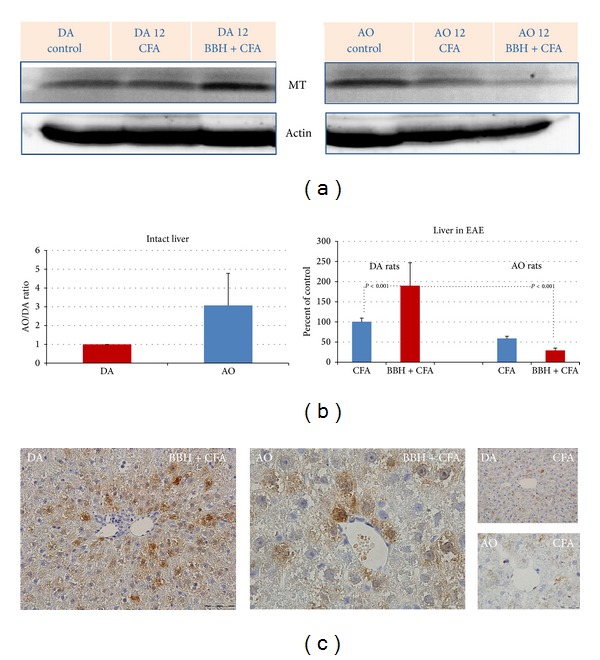
Expression of MT I+II proteins in the livers of EAE-prone DA and EAE-resistant AO rats. (a) Representative western blots indicate the MT I/II protein (~14 kD; range 10–15 kD) detected by anti-MT MoAbs. The liver samples were obtained from intact rats and from rats treated by bovine brain homogenate (BBH) and complete Freund's adjuvant (CFA) or by CFA at the time of first attack in EAE-prone rats (day 12). As a loading control the blots of *β*-actin protein (37–50 kD) are shown. (b) Relative protein expression is shown as AO/DA ratio (intact liver) and as the percent of control (liver in EAE). All band densities were normalized corresponding to *β*-actin. Analyses were made in tissue samples, prepared as a pool from 3 rats in three independent experiments. Data represent mean ± SE. (c) Representative immunohistochemical staining of MT I/II proteins in paraffin-embedded sections of the liver tissue in DA and AO rats after treatment with BBH+CFA or CFA. The results are representative findings of 3 rats.

The data have shown that the EAE-prone DA rats react on immunization with BBH+CFA with a significantly greater increase of hepatic MT I/II proteins than EAE-resistant AO rats (Figures 1(a) and 1(b); *P* < 0.001). However, the content of MTs in intact livers of AO rats was 3 times greater than that in DA rats, pointing to high constitutive expression of these metal-binding proteins. As shown by immunohistochemistry (Figure 1(c)), the immunized DA expressed a higher cytoplasmic and nuclear MT I/II immunoreactivity in hepatocytes than AO rats.

### 3.2. Hepatic Expression of Glycoprotein gp96 in EAE-Resistant and EAE-Prone Rats

**Figure 2:**
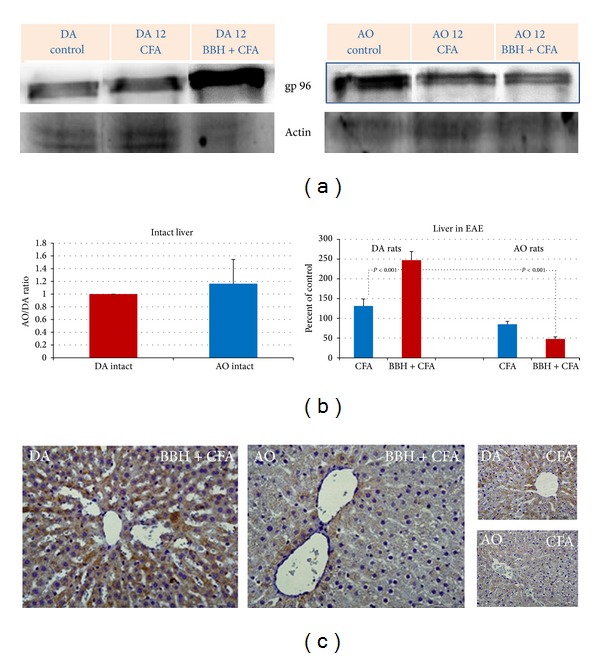
Expression of gp96 in the livers of EAE-prone DA and EAE-resistant AO rats. (a) Representative western blots indicate the gp96/GRP94 protein (~200 kD; range 150–200 kD), detected by MoAb to Grp94/gp96 (clone 9G10) under a nonreducing condition in SDS-PAGE. Liver samples were obtained from intact rats and from rats treated by bovine brain homogenate (BBH) and complete Freund's adjuvant (CFA) or by CFA at the time of first attack in EAE-prone rats (day 12). As a loading control the blots of *β*-actin protein (37–50 kD) are shown. (b) Relative protein expression is shown as AO/DA ratio (intact liver) and as the percent of control (liver in EAE). All band densities were normalized corresponding to *β*-actin. Analyses were made in tissue samples, prepared as a pool from 3 rats in three independent experiments. Data represent mean ± SE. (c) Representative immunohistochemical staining of gp96 protein in paraffin-embedded sections of the liver tissue in DA and AO rats after treatment with BBH+CFA or CFA. The results are representative findings of 3 rats.

The expression of ER-resident HSP-gp96 protein significantly arose only in DA rats, immunized with BBH+CFA (Figures 2(a) and 2(b)). The values were significantly greater that those found in DA rats treated with CFA, as well as than those found in identically treated AO rats (*P* < 0.001). Constitutive expression of gp96 protein in intact livers of DA and AO rats was, however, of similar intensity (Figure 2(b)). High hepatic upregulation of gp96 protein in DA rats was also confirmed by immunohistochemistry, showing prominent cytoplasmic gp96 immunoreactivity in numerous hepatocytes (Figure 2(c)).

### 3.3. Hepatic Expression of IL-6 in EAE-Resistant and EAE-Prone Rats

**Figure 3:**
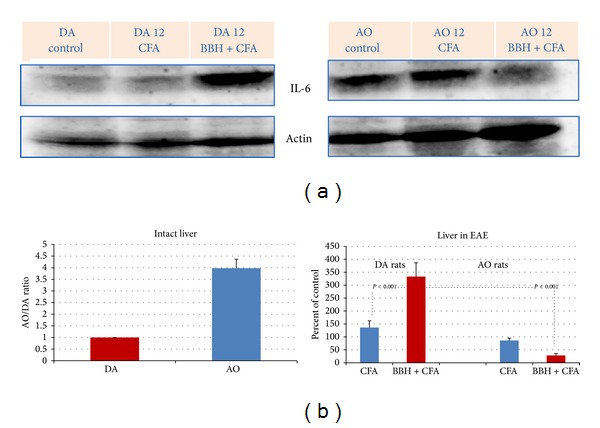
Expression of IL-6 in the livers of EAE-prone DA and EAE-resistant AO rats. (a) Representative western blots indicate the IL-6 protein (~25 kD; range 20–25 kD), detected by mouse anti-rat antibody to IL-6. The liver samples were obtained from intact rats and from rats treated by bovine brain homogenate (BBH) and complete Freund's adjuvant (CFA) or by CFA at the time of first attack in EAE-prone rats (day 12). As a loading control the blots of *β*-actin protein (37–50 kD) are shown. (b) Relative protein expression is shown as AO/DA ratio (intact liver) and as the percent of control (liver in EAE). All band densities were normalized corresponding to *β*-actin. Analyses were made in tissue samples, prepared as a pool from 3 rats in three independent experiments. Data represent mean ± SE.

Similarly, in the livers of EAE-prone DA rats we found a high upregulation of hepatic IL-6 protein during the first attack of disease. Its values in livers of immunized DA rats were significantly higher than those in DA rats treated by CFA, as well as than those in EAE-resistant AO rats (Figures 3(a) and 3(b); *P* < 0.001). However, resembling the findings for MT I/II, in the livers of intact AO rats the expression of IL-6 was four times higher than that in intact DA rats.

### 3.4. Hepatic Expression of TGF-*β* in EAE-Resistant and EAE-Prone Rats

**Figure 4:**
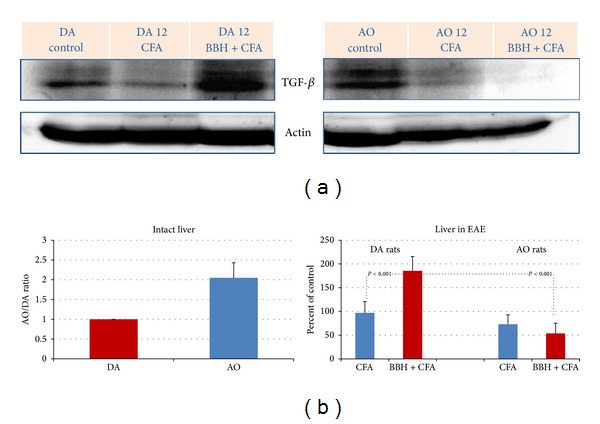
Expression of TGF-*β* in the livers of EAE-prone DA and EAE-resistant AO rats. (a) Representative western blots indicate the TGF-*β* protein (~44 kD; range 35–55 kD), detected by mouse anti-rat antibody to IL-6 in the liver samples obtained from intact rats and rats treated by bovine brain homogenate (BBH) and complete Freund's adjuvant (CFA) or by CFA at the time of first attack in EAE-prone rats (day 12). As a loading control the blots of *β*-actin protein (37–50 kD) are shown. (b) Relative protein expression is shown as AO/DA ratio (intact liver) and as the percent of control (liver in EAE). All band densities were normalized corresponding to *β*-actin. Analyses were made in tissue samples, prepared as a pool from 3 rats in three independent experiments. Data represent mean ± SE.

Hepatic expression of TGF-*β* was found to be similar to that of MT I/II and IL-6. Its values became markedly upregulated during the first attack of disease only in DA rats (Figures 4(a) and 4(b); *P* < 0.001). Besides, the constitutive expression of TGF-*β* in livers of intact AO rats was 2 times greater than that in DA rats (Figure 4(b)).

## 4. Discussion

Using an animal model, which in DA rats induces the clinical symptoms of disease that resemble to relapsing-remitting form of MS in humans, we show herein that hepatic IL-6, TGF-*β*, MTs, and ER-resident HSP-gp96 have a high regulatory effect on inflammation and disturbed immune mechanisms that occur during an autoimmune response. Besides, the data imply that the susceptibility to EAE might be in association with the constitutive expression of hepatic MT I/II, which are the essential cytoprotective proteins involved in the regulation of different types of injuries. The cellular and molecular mechanisms remain to be elucidated, but the hypothesis is consistent with the well-known function of the liver in controlling acute phase response [25] and both immunity and tolerance [17, 26, 27]. In this context, it has been repeatedly shown that the liver is characterized by a specific hepatic microenvironment, created by glucocorticoids (GCs), local cytokines [5], and the unique hepatic APC populations, which results in induction of the antigen-specific peripheral tolerance rather than in induction of T-cell immunity [17, 21]. In addition, it is known that, during an immune response, a wide variety of mediators, including cytokines, growth factors, and hormones, must control also the concomitant liver injury, inflammation, and repair activating in different populations of liver cells (hepatocytes, stellate cells, Kupffer cells, sinusoidal endothelial cells, and lymphocytes) and various downstream signaling pathways. The response depends on interactions of various mediators, on the activation of individual Janus kinase (JAK)-signal transducer and activator of transcription (STAT) pathways, on interplay between the STAT1 and STAT3 activation, and on subsequent activation of cytokine-induced negative feedback loop that terminates JAK-STAT signaling by activation of suppressors of cytokine signaling, SH2-containing phosphatases, and protein inhibitors of activated STATs. Owing to this, the activation of various STATs might give anti- or proinflammatory signals, depending on the STATs activated, on the cell types in which the STATs are activated, and on the type of liver disease or liver injury that has been studied (reviewed by [28]).

In this regard, it has been shown that IL-22, IL-6, and IL-6 family of cytokines led in hepatocytes and myeloid cells to STAT3 activation, which acts as an anti-inflammatory signal that suppress liver inflammation. In contrast, the activation of STAT3 in T cells may even promote liver inflammation, since it leads to the upregulation of the ROR*γ*t and ROR*α* transcription factors that promote differentiation of T cells towards a Th17 phenotype, contributing to the IL-17 production. Moreover, the latter is highly influenced by balance of IL-6 and TGF-*β*, since IL-6 may inhibit TGF-*β* induced differentiation of immunosuppressive Treg and induce, in combination with TGF-*β*, the development of proinflammatory Th17 cells from naıve T cells [29]. The issue is covered by excellent reviews in this field [30–33], showing that cytokines estimated in our study may have high impact on self-tolerance and development of autoimmunity.

Consistent with this proposal in the present study we show that IL-6 and TGF-*β* arose only in the livers of EAE-prone DA rats during the first attack of EAE (Figures 3 and 4) implying that hepatic IL-6 and TGF-*β* contributed to the generation of antigen-specific aggressive TH17 or TH1 cells. However, in these considerations the time-course of events should be taken into account, since IL-6 participates also in processes that arrest the inflammatory response to infection and tissue injury and ensure a good restoration of the affected area. In this sense, it was shown that IL-6 stimulates the transition from innate to acquired immunity [34], acting as a factor that limits the entry of neutrophils into the affected area and induces their elimination by apoptosis and as a factor that stimulates the entry of monocytes and enables the survival of T cells controlling the expression of several chemokines and cytokines and adhesion molecules of the vascular endothelium (reviewed by [35]). Furthermore, inducing in the liver the synthesis of type II acute phase proteins (fibrinogen, haptoglobin (human), *α*1-antichymotrypsin, *α*1-antitrypsin, and *α*2-macroglobulin) IL-6 may limit the proteolytic and/or fibrogenic activity and tissue damage [25, 36]. In addition, IL-6 and other proinflammatory cytokines, such as TNF*α*, IL-1*α*, and IL-1ß, may lead to the activation of the hypothalamic-pituitary-adrenal (HPA) axis feedback circuit, which increases the levels of endogenous glucocorticoids (GCs), as the immunosuppressive end product of this pathway [5, 6]. Besides, it should be emphasized that IL-6 is a critical cytokine, which induces the transcription of MT I/II both in the liver [37–39] and in the brain [35, 40], leading eventually to the marked cytoprotective and neuroprotective outcome, as it has been shown in various physiological conditions [16, 41] and in EAE [42], MS, [43] and other types of brain injury [13, 44, 45]. Accordingly, IL-6 deficiency may increase the oxidative stress during CNS inflammation [40]. However, since the overproduction of IL-6 and abnormalities in IL-6 signal transduction might be causative factors in several autoimmune disorders, the IL-6 blockade might be an effective approach in their treatment (reviewed by [33]). In line with this evidence, it was shown that anti-IL-6R antibodies, administered immediately after immunization of mice with myelin oligodendrocyte glycoprotein (MOG) peptide, were able to suppress the occurrence of EAE, leading to elimination of the Th17 cells in the draining lymph nodes and in the spinal cord [46].

Supporting the current knowledge about the prominent regulatory functions of metallothioneins in pathogenesis of EAE and other types of brain injuries [35, 45, 47], we previously reported that the expression metallothioneins I/II might be induced in both EAE-prone and EAE-resistant strain of rats, even in the presymptomatic phase of CR-EAE (on the seventh postimmunization day) in cells that form blood-brain and blood-cerebrospinal fluid barriers, in the cerebellar parenchyma and hippocampal dentate gyri, as well as in the liver of rats [12]. However, on the 12th postimmunization day, the levels of hepatic IL-6, TGF-beta, MTs, and gp96 were significantly greater in DA than in AO rats, implying that AO rats might better control the autoimmune reaction [12]. Moreover, since in AO rats we noticed an early upregulation of TGF-*β* on several hepatic structures (vascular endothelium, Kupffer cells, and hepatocytes), we speculated that specific hepatic microenvironment might contribute to the faster recovery of these rats from EAE [12]. The new data from current study, also revealed that AO rats have greater basal levels of TGF-*β*, IL-6, and MT I/II (Figures 4, 3, and 1), implying that the susceptibility in AO and DA rats might depend not only on MHC haplotype, but also on some epigenetic traits that might be responsible for immunomodulation [48].

Our data do not permit further discussion in this direction since the susceptibility to EAE is a polygenic trait, in which small differences in the expression or activities of different gene products combine to determine the initiation of disease [1–3]. In most strains the response is governed by the MHC class II gene products and by the T-cell receptor (TCR) repertoire, which recognize the dominant encephalitogenic epitope, but the susceptibility may depend also on genetic traits responsible for interindividual differences in metabolism and sensitivity to various pathogens and endogenous factors. In addition, it often correlates with dominant type-1 (cell-mediated) or type-2 (humoral or antibody-mediated) immunity of the strain, which are highly influenced by neuroimmune interactions and signaling within the hypothalamic-pituitary-adrenal axis [49], as well as by regulatory mechanisms that include the generation of cells and cytokines with suppressive activity and activation of the pathways that ensure the apoptotic elimination of autoaggressive T-cell clones [5, 6].

As a small segment of this complex network, we show herein that in regulation of susceptibility to EAE participate also hepatic MTs, owing to their important functions in metal ion homeostasis and redox control and in protection against heavy metals, DNA damages, stress, and inflammation [16, 41, 50, 51]. Moreover, owing to their ability to control Zn-dependent transcription factors, protein synthesis, cellular energy, levels and metabolism, they are also critically involved in cell growth and multiplication [37, 52], as well as in the maintenance of neuroimmune homeostasis and processes of apoptosis [51]. Accordingly, we can speculate that high constitutive MT expression in AO rats and high inducible hepatic MTs in DA rats contributed to lower susceptibility to EAE and to the restrain of autoimmune and inflammatory response. It is likely that the underlining mechanisms are different in quiescent and injured liver tissue, but at the present they remain elusive. However, reflecting the antioxidative activity of MT I/II during EAE, we previously reported that in DA rats tissue content of free zinc ions increased at the sites of MTs induction [24] as well as that during the second attack of CR-EAE (on the 22nd postimmunization day) the hepatic expression of MT I/II was accompanied by high accumulation of Zn and Cu in the liver [23]. Moreover, linear regression analysis made in the present study revealed that in the intact liver of AO rats the tissue levels of MTs were in high positive correlation with tissue levels of IL-6 (*r* = 0. 87). In contrast, in immunized rats of both strains hepatic IL-6 (detected on 12th day after immunization with BBH+CFA) was in a high negative correlation with the hepatic gp96 (*r* = 0.97). However, these preliminary data need to be confirmed (not shown).

Furthermore, in this study we show that during EAE markedly changes also the hepatic expression of ER-resident HSP-gp96 (Figure 2), confirming the contribution of endogenous DAMP signals in development of autoimmune response [4, 19]. Owing to its chaperon role in the unfolded protein response (UPR) [53–55] and ER-associated degradation (ERAD) [56] there is a possibility that hepatic gp96 during EAE was involved in the processes that regulate the proper folding and assembly of newly synthesized secretory and membrane proteins and in those that prevent the aggregation of unfolded and incompletely folded proteins in the ER. Besides, owing to its ability to actively chaperon MHC class I-restricted epitopes into the cross-presentation pathway of professional antigen-presenting cells (APC) and induce the activation and maturation of these cells [19, 57], we can also speculate that gp96 actively contributed to the induction antigen-specific immunity after the immunization with BBH-CFA. The hypothesis is confirmed by numerous reports pointing to a high upregulation of HSP in CNS during the exposure of cells to various kinds of stress and infection [58, 59], as well as in MS [60], EAE [61], and other neurological diseases [18], often called “chaperonopathies.”

Direct effects of gp96 on EAE is also confirmed by finding that immunization of mice with HSP+peptide complexes might elicit CD8+ and CD4+ T-cell responses specific to the HSP-chaperoned antigenic peptides [19] as well as that immunization with high doses of gp96 might prevent myelin basic protein or proteolipid protein-induced autoimmune encephalomyelitis in SJL mice by induction of suppressor CD4+ population, showing that gp96 might induce both the antigen-specific activation or suppression of cellular immune responses [62].

Confirming that gp96 might be involved in proteostasis and immune-related pathways, linked with the reparative processes in the CNS, we recently reported that in DA rats the constitutive gp96 expression, found in several neurons and glial cells in the brain and spinal cord of intact animals, significantly diminished during the attacks of CR-EAE as well as that gp96 was upregulated during the remission phase of CR-EAE disease in the oligodendrocytes, in the neurons of the hippocampal area, and in the motoneurons of lumbar spinal cord [22]. The finding points to the role of gp96 in immune mechanisms showing that gp96 may stimulate the Treg cells in a dose dependent manner [63] as well as that gp96 may regulate the generation of tolerogenic pDCs [64] and participate in maintenance of tolerance in multiple sclerosis [65].

We would like also to underline that in other animals models with disturbance of morphostasis (liver regeneration, stress, syngeneic pregnancy, treatment with peptidoglycan, and autoimmune diabetes) [66, 67] the hepatic gp96 overexpression often correlated with a high accumulation of natural killer (NKT) cells, as well as with upregulation of costimulatory molecule on APC and generation of Treg in the liver and in the thymus, pointing to the general role of ER chaperons in the maintenance of immune homeostasis and morphostasis [66, 67]. Based on these lines of evidence and current knowledge of immune functions of gp96 [20] we can, therefore, speculate that hepatic gp96 during EAE might affect the maturation of local dendritic cells and their tolerogenic and cytokine induction potential, as well as the functions of hepatic NKT cells, which recognize the conserved stress-induced self-structures, rather than variable foreign antigens [68]. However, although it was shown that the liver-confined invariant NKT cells after activation by alphaGalCer might suppress Th1-cytokine production and foster the secretion of IL-10 from MOG35-55-specific T cells [69], our data need further experiments in this direction.

Concluding, we would like to underline that the expression profile of MTs and gp96 in DA rats immunized with BBH+CFA showed a large temporal and regional variability [23], emphasizing their interactions with multiple control pathways. In our experiments the MT I/II expression correlated more with the peaks of clinical symptoms, in contrast to gp96, which was found in remission phases, suggesting that ER chaperones might be induced to ameliorate the accumulation of misfolded proteins in the CNS and protect neuronal cells against autoimmune attack. The importance of time-course evaluation of the data is emphasized also in the present study, which imply that EAE-resistant rats may more efficiently restrain the autoimmune response, owing to greater constitutive expression of hepatic MT I/II and IL-6 (Figures 1 and 3) and owing to an early upregulation of TGF-*β* in the liver [12].
